# Regular Exposure to Cowbells Affects the Behavioral Reactivity to a Noise Stimulus in Dairy Cows

**DOI:** 10.3389/fvets.2017.00153

**Published:** 2017-09-29

**Authors:** Julia Johns, Sophie Masneuf, Antonia Patt, Edna Hillmann

**Affiliations:** ^1^Faculty of Organic Agricultural Sciences, Farm Animal Behavior and Husbandry Section, University of Kassel, Witzenhausen, Germany; ^2^Ethology and Animal Welfare Unit, Department of Environmental Systems Science, Institute of Agricultural Sciences, ETH Zurich, Zurich, Switzerland; ^3^Department of Neurology, University Hospital Zurich, Zurich, Switzerland; ^4^Institute of Animal Welfare and Animal Husbandry, Friedrich-Loeffler-Institut, Federal Research Institute for Animal Health, Celle, Germany; ^5^Animal Husbandry, Albrecht Daniel Thaer-Institute of Agricultural and Horticultural Sciences, Humboldt-Universität zu Berlin, Berlin, Germany

**Keywords:** avoidance, behavior, cattle, earplug, noise, playback

## Abstract

In alpine regions, cows are often equipped with bells during pasture season to ensure that farmers can locate them. Constant exposure to the chime of a bell may affect cows’ acoustic perception in general. The aim of this study is to test whether routine bell exposure affects the reactivity to a noise stimulus and might be associated with hearing impairment in cows. For the assessment, behavioral and cardiac indicators were used as indirect measures of hearing capacity. Cows that were either used to wearing a bell or not were exposed to a playback of low and high amplitude (=varying loudness). In addition, we tested whether wearing earplugs, mimicking hearing impairment, reduced the cows’ reactivity toward the playback. On 24 farms, half of them routinely using cowbells, 96 Brown Swiss cows were tested in a 2 × 2 factorial cross-over design (65 or 85 dB, without or with earplugs) in a balanced order. The effects of bell experience, amplitude, and earplugs on the latency to the first behavioral and cardiac response to a 5-s playback were analyzed using linear mixed-effects models, considering dependencies within the data set. Cows reacted faster without earplugs and when they were exposed to 85 dB compared with 65 dB. The proportion of cows leaving the feeding rack after onset of the playback was reduced by bell experience and earplugs and was increased when exposed to 85 dB compared with 65 dB. Exposure without earplugs to 85 dB but not to 65 dB increased heart rate. Heart rate and heart rate variability indicated increased sympathetic activation during the exposure to 85 dB compared with 65 dB. In general, behavioral and cardiac indicators did not indicate severe hearing impairment due to routine bell exposure. The 85-dB stimulus increased arousal and avoidance compared with the 65-dB stimulus, with bell experience and earplugs leading to a general decrease in avoidance of the stimulus. This may reflect an altered acoustic perception of the playback stimulus in dairy cows that are routinely exposed to bells.

## Introduction

In alpine regions, cows are often equipped with a bell throughout the summer season to ensure that farmers can locate their animals on the wide alpine pastures, many areas that are obstructed from view. The chime of these cowbells is characterized by high and varying amplitudes from 90 to 113 dB at a distance of 20 cm, the approximated distance between the bell and the cows’ ears (1). Goats have been found to show higher behavioral arousal when being exposed to the playback of a bell compared to the playback of a uniform sinusoidal sound, indicating that the bell sound might be more aversive to goats than the uniform sound. With repeated exposure, goats habituated to both stimuli (2).

So far, little research has been conducted investigating the effect of noise on the hearing capacities of animals. Kenneled dogs that were constantly exposed to noise between 100 and 108 dB for 6 months developed hearing loss as indicated by measurements of the auditory brainstem response (ABR) (3). In mice, ABR recordings showed that a single exposure to noise of 100 dB for 2 h induced temporary hearing loss (4), and an exposure to noise of 110 dB for 60 min even induced permanent hearing loss (5).

Noise-induced hearing loss is one of the most common causes of exogenously acquired sensorineural hearing loss in adult humans (6). Although anatomic differences among mammal species lead to differences in hearing capacities (7), the basic physiologic processes underlying the detection and sensation of sound are essentially identical between humans, dogs, cattle, and mice (8–11). Considering that cows can hear sounds between 23 Hz and 35 kHz, with the highest sensitivity at 8 kHz, and are able to detect sounds at −11 dB, i.e., amplitudes the human ear cannot detect (11), the continuous exposure to bells during pasturing season might impair the cows’ hearing capacity.

Behavioral indicators such as the acoustic startle response (12–15) or avoidance reactions (16, 17) have been used as an indirect but non-invasive test of hearing capacity in earlier studies. The acoustic startle response is an electromyographic response, which in rodents is elicited by stimuli with an amplitude of more than 80 dB (18, 19), and the latency is very short [5–10 ms for the electromyographically measured response in different muscles (20–22)]. Behaviorally, a startle response is defined as a cross-species response to an intense and abrupt stimulus (23) and as any first reaction of any part of the body, such as body movements, movements of limbs and facial movements, or any first behavioral reaction to sound stimulation (24). Therefore, the latency to the first behavioral reaction, e.g., sudden head movements in response to an acoustic stimulus can be used as a proxy for the induction of a startle response. In addition, avoidance reactions in response to an acoustic stimulus, e.g., increasing the distance between the source and oneself, indicate that the stimulus is perceived as aversive by the animals (25–27).

In addition, cardiac parameters such as heart rate and heart rate variability can be used to assess arousal induced by noise (2, 28). If cardiac parameters indicate an arousal due to noise exposure, it can be assumed that the animal perceives the noise as aversive (23). Noise exposure is often accompanied by an increase of heart rate in humans (29, 30). Lee et al. (31) evaluated instant responses of the autonomic nervous system to short-duration noises using heart rate variability analysis. The results indicated that, compared with background noise of 38 dB, exposure to noise between 50 and 80 dB increased sympathetic activity as indicated by a higher ratio of low-frequency (LF) to high-frequency (HF) spectral power. For humans, “hazardous” noise is defined as sounds that exceed 85 dB over a typical 8-h workday (32, 33). It has been shown that constant exposure to such hazardous noise can result in irreversible hearing loss and that even a single intense sound event can cause hearing loss and tinnitus (32, 33).

A widespread solution used to protect humans and also horses from noise exposure by using hearing protection devices such as earplugs (34, 35). Commercially available earplugs for horses are made of memory foam (35). In cattle, acoustic earphones were inserted into the ear canal and were held in position with either silicone earplugs or earplugs of compressible foam while measuring brainstem auditory evoked potentials (BAEPs) (36–38). Such earplugs occlude the ear canal and attenuate background noise.

Although some studies on the general hearing capacity of cows are available, to our knowledge, no studies exist on hearing capacities of cows that have been exposed to noise in general. Bells seem to be a relevant noise factor for cows considering that cows are exposed routinely and for a longer period of time to the chime of bells (1). The aim of this study is to test whether routine bell exposure affects the reactivity to a noise stimulus and be associated with hearing impairment in cows. Behavioral and cardiac indicators were used as indirect measures for the assessment of cows’ hearing capacity. Thus, we examined the reactivity toward noise of low (65 dB) and high (85 dB) amplitude in bell-experienced and bell-inexperienced cows on 24 Swiss dairy farms. We additionally tested whether mimicking hearing impairment using earplugs would reduce the reactivity to the sounds. We hypothesized that cows that had been exposed regularly to a bell on alpine pastures (bell-experienced cows) would show reduced reactivity toward these sounds (increased latency of the first behavioral reaction, reduced avoidance and heart rate, and increased heart rate variability) contrarily with cows that were only equipped with a bell as heifers or never before (bell-inexperienced cows). Contrary, we expected that cows would show increased reactivity in response to a stimulus of high amplitude (decreased latency of the first behavioral reaction, increased avoidance and heart rate, and reduced heart rate variability) compared to a stimulus of low amplitude. Further, we expected that cows without earplugs would also show increased reactivity toward these sounds (decreased latency of the first behavioral reaction, increased avoidance and heart rate, and reduced heart rate variability) compared to cows with earplugs. Altogether, if earplugs do not diminish the reaction of a given cow, this might be an indicator of either a low-reactive animal, well habituated to noise or hearing impairment.

## Animals, Materials, and Methods

Ethical approval to conduct the study was obtained from the Zurich Cantonal Veterinary Office, Switzerland (approval No. 77/2012).

### Animals, Housing, and Management

The study was performed between September and November 2013 on 24 Swiss farms, with 96 multiparous Brown Swiss cows that were between 3 and 10 years of age. The owners of the cows gave permission to conduct the study on their farms. The size of the farms varied between 8 and 100 animals. On each farm, four experimental cows were selected randomly. On 12 farms, cows were used to wearing a bell either every year for 4–5 months during the summer season or all year round (48 cows on 12 farms, “experienced”). On the other 12 farms, cows had no or very little experience with wearing a bell, having either never worn a bell before or only once for 4–5 months when they were 1 year old (48 cows on 12 farms, “inexperienced”). On all 24 farms, cows were kept in cubicle housing systems with a feeding rack and headlocks. All cows were familiar with being locked in the feeding rack during feeding. They were fed with hay, fresh grass or a mixed ration of hay, corn, and grass silage. Feed and water were provided *ad libitum*. The cows were milked twice a day.

### Test Area within Barn

**Figure 1:**
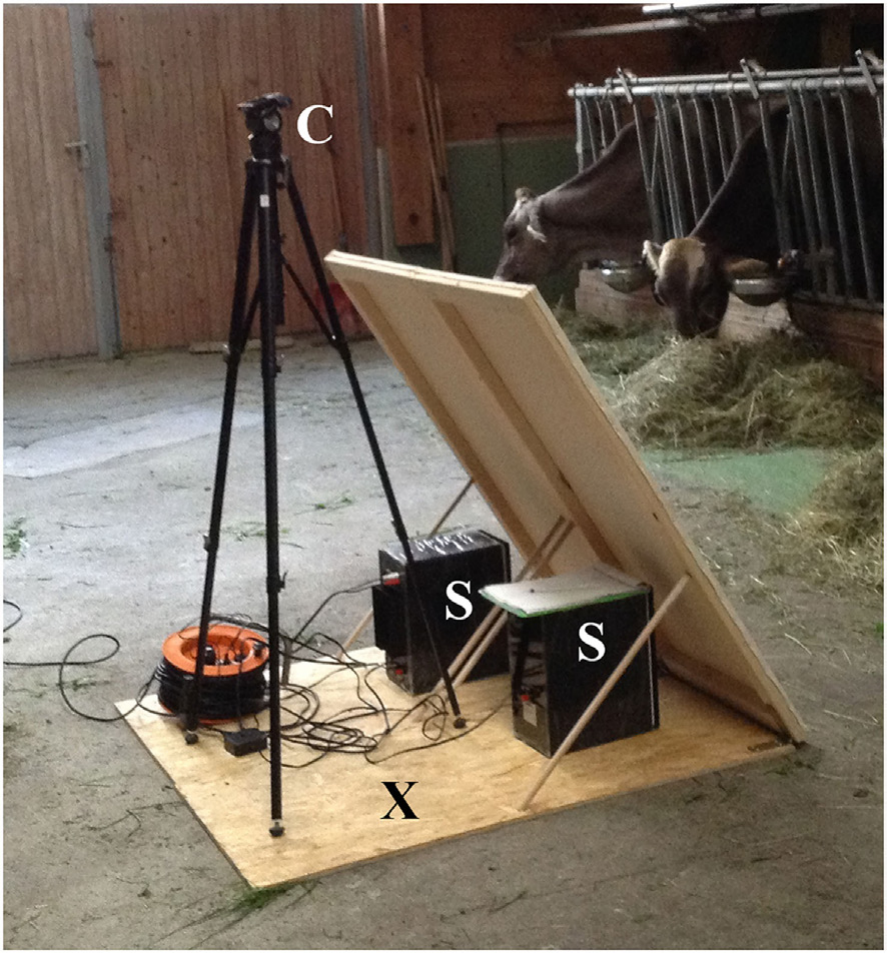
Technical equipment. Technical equipment behind a visual cover of white fabric. C, camera; S, speakers; X, experimenters were hidden behind the visual cover during playback.

The experiment was carried out at the feeding rack of each farm during the course of 1 day. A 5-m section of the feeding rack served as test area for the experiment. During the experiment, only the four experimental cows had access to this area, one cow at a time. To record the animals’ behavior, a video camera with an integrated microphone (Canon^®^ Legria FS 200 digital camera) was mounted on a tripod and positioned in front of the separated feeding rack area (Figure 1). The acoustic stimulus was transmitted *via* two loudspeakers (Edifier^®^ S2000v, Edifier International, Hong Kong, China) that were positioned on the floor next to the camera. The loudspeakers, the tripod, and cables were hidden behind a visual cover of white fabric (Figure 1) and placed on a board (approximately 1.5 m × 1.5 m). The front edge of the board with the loudspeakers was placed in the feeding alley at a distance of 2.15 m from the feeding rack to ensure that the intended amplitude reached the cows’ ears.

### Acoustic Stimulus

**Figure 2:**
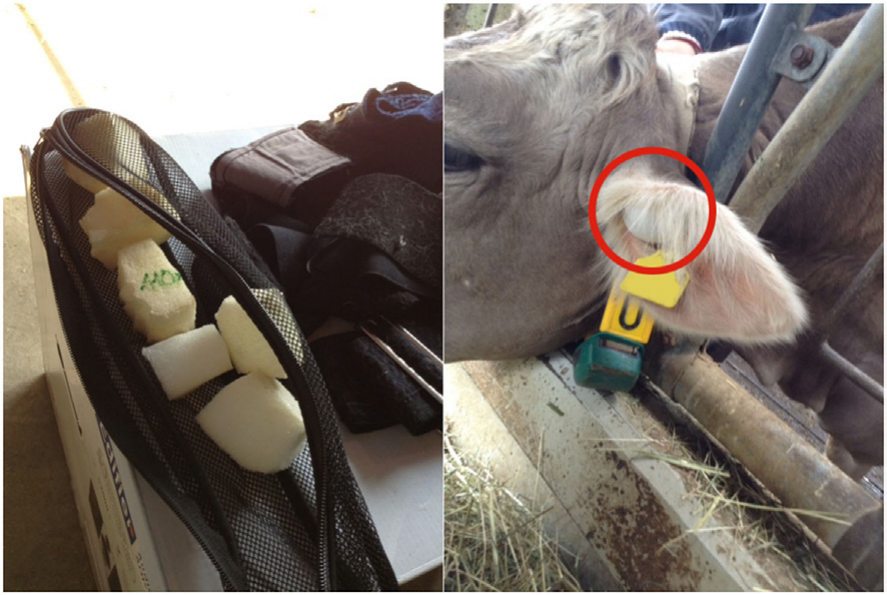
Earplugs. Cow with earplug (70 mm × 40 mm × 40 mm) made of memory foam (left) in the ear canal (right).

Each cow was exposed to a pink noise stimulus broadcast four times for 5 s in a 2 × 2 factorial design: each cow was tested with and without earplugs and at 65 (A-weighting scale, A) and 85 dB (A) (Figure 2; each amplitude with two phases: without and with earplugs), i.e., each of the bell-experienced and bell-inexperienced cows were exposed to each amplitude (65 and 85 dB) twice, once with and once without earplugs (4 trials per cow, see Experimental Procedure). The A-weighting scale assigns low weights to the LF tones, to which the human ear and the ears of some animals are less sensitive, and high weights to the HF tones, to which humans are more sensitive (39).

Pink noise is characterized by uniformly distributed energy throughout the range of human hearing, approximately 20 Hz–20 kHz. Most people perceive pink noise as having uniform spectral power density, i.e., the same loudness at all frequencies (40). The noise stimulus was automatically generated by a Tone Generator Pro v1.0.8 (Performance Audio^®^) for iPhone^®^. We chose the pink noise for three reasons: first, it has no biologic relevance for the cows, and therefore, the reactions to the acoustic stimuli were mostly likely limited to the perception of the acoustic stimuli *per se*. Second, it contains all frequencies from 20 Hz to 20 kHz at the same amplitude and should thus match the cows’ (potential) hearing capacity. Third, it was a novel acoustic stimulus for all cows, and none of them was ever exposed to it before. The volume of the playback was set at a level that ensured an amplitude of either 65 or 85 dB (A) at the feeding rack at approximately 60 cm above the floor (i.e., the estimated position of the cows’ heads when feeding). The amplitude was measured with a precision noise level measuring instrument with integrated long-term storage (SoundTest-Master, Laserliner^®^, Umarex GmbH & Co. KG, Arnsberg, Germany). The background noises at the farms measured before the start of the experiments ranged from 40 to 60 dB (A).

### Experimental Procedure

**Figure 3:**
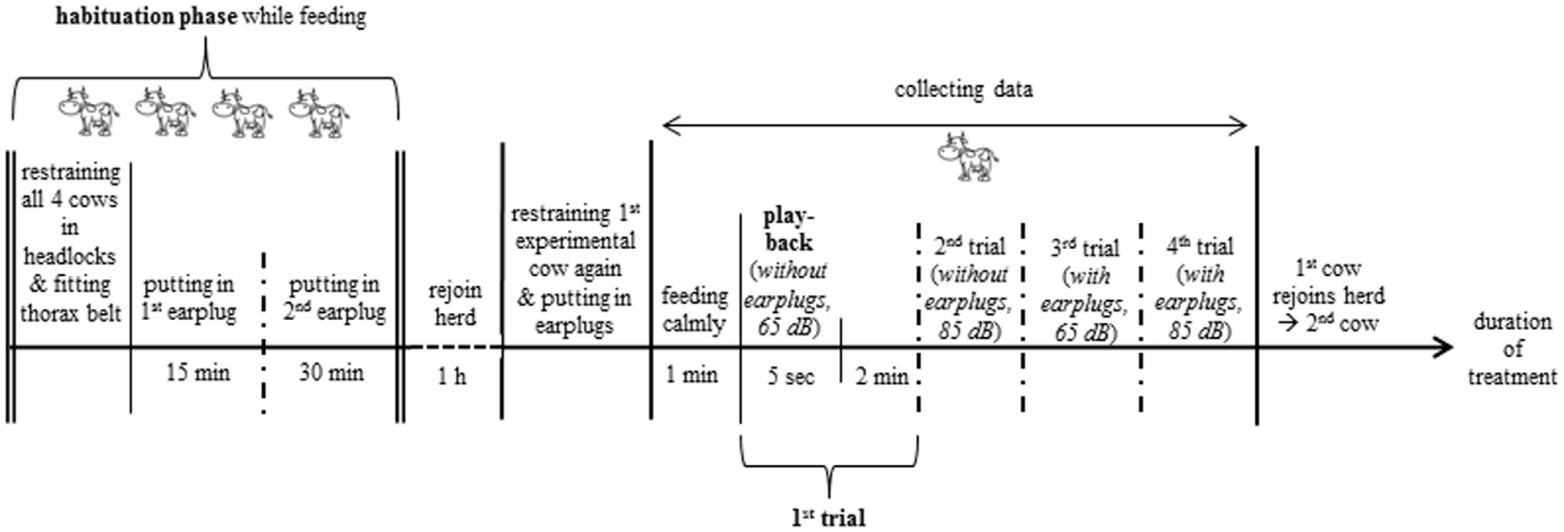
Experimental procedure. The two playbacks (65 and 85 dB) with earplugs were tested in consecutive trials; the order of amplitude within a phase (without or with earplugs) and between phases was chosen randomly for each cow.

In the morning around 8 O’clock, the visual cover of white fabric with all technical equipment was set up. Before the start of the experiment, the four experimental cows were habituated to the technical equipment (Figure 1), the thorax belt to measure heartbeat parameters (see Heartbeat Measurements) and the earplugs (Figure 2). Each experimental cow was exposed to the pink noise stimulus in four trials (Figure 3). The order in which the cows were tested was chosen randomly before the start of the experiment. To reduce handling of the cows, the two playbacks during which cows were equipped with earplugs were tested in consecutive trials. Thus, each cow was equipped with the earplugs only once during the experiment with the order of phases and amplitudes chosen randomly for each animal.

On each farm, the four experimental cows were led to the separated 5-m section of the feeding rack, restrained in the headlocks (for feeding), and fitted with a thorax belt. Then, they were habituated to the earplugs. The earplugs (70 mm × 40 mm × 40 mm; Figure 2) were made of memory foam (polyurethane with additional non-toxic chemicals increasing its viscosity and density; Vibraplast AG^®^, Aadorf, Switzerland). Earplugs were compressed and placed into the ear canal during feeding, where they expanded and plugged the ear canal. To avoid experimental cows being irritated by a sudden impairment of hearing, the first earplug was positioned into the left ear for 15 min before the second earplug was placed into the right ear. Experimental cows were left undisturbed for 30 min with both thorax belt and earplugs in place. During these 30 min, the two earplugs had to remain in position for at least 15 min. If the animal shook its head resulting in the loss of an earplug, the earplug was repositioned. During the first 15 min, the first earplug had to be repositioned 1.3 times (minimum 1 and maximum 6 times), and during the following 30 min, an earplug had to be repositioned 1.9 times (minimum 1 and maximum 7 times). At the end of the habituation phase, all experimental cows accepted the earplugs and continued to feed calmly. After the habituation phase, the earplugs were removed, and the experimental cows were allowed to rejoin the herd for 1 h. To ensure that experimental cows were motivated to feed, cows had no access to feed while rejoining the herd.

After the 1-h break, experimental cows were individually led into the separated area of the feeding rack, restrained in the headlocks, and fitted with the earplugs depending on stimulus order (Figure 3). To avoid exposure to the acoustic stimuli prior to testing, the other experimental cows were either separated in an outdoor paddock (*n* = 11 farms) or fitted with earplugs and led to the farthest part of the stable (*n* = 13 farms).

A handful of concentrate was sprinkled on the usual feed during trials to enhance feeding motivation of the experimental cow. As soon as the experimental cow was feeding calmly for at least 1 min, the headlocks were carefully opened by the second experimenter, and the playback was switched on for 5 s (20 ± 3 s after opening the headlocks). Start and stop of the playback were controlled manually by the experimenter using a mobile phone (iPhone 4 s^®^, Apple Inc.). After the end of the playback, behavioral observations and heartbeat measurements continued for 2 more minutes (playback + 2 min = trial). Each experimental cow was tested in all four trials in one session. When the session was finished, the thorax belt (and earplugs, depending on phase order) was removed, and the cow was allowed to rejoin the herd. If a cow did not start feeding within 1 min after offering the concentrate, no playback was conducted, and another experimental cow was tested.

### Behavior

Behavioral analyses were conducted by two different people (person 1 analyzed the latency to the first reaction, and person 2 analyzed avoidance) who had not participated in the conduct of the experiments. They did not know the farms and were not aware of the aim of the study. However, as they needed to record the behaviors related to the start of the playback, they were aware that acoustic stimuli were involved but were blind to the bell experience of the animals, the amplitude, or if the animals were equipped with earplugs.

In addition, to test intraobserver reliability for the assessment of latency to the first reaction, person 1 assessed 96 trials twice.

#### Latency to the First Reaction

The latency to the first reaction was described as time (seconds) it took the experimental cows from the onset of the playback to react with a clearly visible change of the ear posture, pausing (cow stopped her current behavior and froze shortly), or a sudden head movement (cow stopped feeding and raised its head quickly). The latency to the first reaction was analyzed in slow motion (0.5× real time) from video using VLC media player^®^ (version 2.2.4 Weatherwax, VideoLAN, Paris, France) and a stopwatch for Smartphone Shift5.1^®^ (Android version 5.1, SHIFT GmbH, Falkenberg, Germany) for 15 s after onset of the playback.

#### Avoidance

An avoidance reaction to the playback was recorded whenever a cow left the feeding rack (completely withdrawing the head from the feeding rack) within 60 s after onset of the playback using INTERACT^®^ (Mangold International GmbH, version 9.0.7, Arnstorf, Germany). A duration of 60 s (=5 s playback + 55 s) as a time window that may reflect a response to the playback and not to any other stimuli, e.g., environmental sounds was considered.

### Heartbeat Measurements

Heartbeat measurements were recorded using Polar^®^ Equine (Polar^®^ Elektro Europe BV, Zug, Switzerland), allowing a non-invasive measurement of heartbeats (41, 42). To increase the electrode–skin contact, electrode gel (Anandic Medical Systems AG/SA, Feuerthalen, Switzerland) was used. A thorax belt with two integrated electrodes was fixed around the torso directly behind the forelegs. One electrode was positioned ventrally on the left side of the sternum and the other one at a given distance by the thorax belt on the left thoracic wall. A receiver for recording the data was placed between the two electrodes. The thorax belt was additionally protected by an elastic belt of about 5 cm width. The heartbeat was recorded for 1 min of continuous feeding before the playback started, during the playback (5 s), and 2 min after the playback. Data were downloaded onto a computer *via* a base station using Bluetooth (Polar^®^ Team^2^ Pro, version 1.3.0.3).

Analysis of cardiac data was carried out using the programs Polar^®^ ProTrainer 5 Equine Edition (version 5.35.161, © Polar Electro Europe AG, Zug, Switzerland) and R 3.2.3 (43). Root mean square of successive differences (RMSSD) of heartbeats reflects changes in the vagosympathetic balance that are vagally mediated (42) and represents parasympathetic activity, whereas standard deviation of heartbeats (SDNN) is a more complex parameter reflecting vagal and sympathetic activation (42, 44). The spectral measures HF band and LF band are highly correlated with the time domain-related measures RMSSD and SDNN, respectively (45, 46). Consequently, the ratio between RMSSD and SDNN can be used as an indicator of changes of the vagosympathetic balance in the organism (47–49), similar to the ratio of HF and LF (44). An increased ratio between RMSSD and SDNN indicates that vagosympathetic balance is more shifted toward parasympathetic activation, whereas a lower ratio indicates a shift toward sympathetic activation (49).

Automatic correction of the tachograms was carried out using the correction routines included in the Polar^®^ software (Polar^®^ ProTrainer 5 Equine Edition, version 5.35.161). Data with an error rate of more than 5% were excluded from the analysis according to the studies by Hagen et al. and Gygax et al. (46, 50). In addition, data of one cow were excluded from analysis due to extremely high heart rate regardless of experimental treatment. This led to the exclusion of 286 trials and a remaining sample size of 98 trials.

The number of R-R intervals [heart rate in beats per minute (bpm)], the RMSSD (ms) and the ratio between RMSSD and SDNN (RMSSD/SDNN) were calculated. The 1 min preceding the playback and the first minute of the trial were both divided into first (0–20 s), middle (21–40 s), and last (41–60 s) 20 s. The middle 20 s of the minute preceding the playback was then chosen as reference value for heartbeat parameters. This reference time window was compared with the first time window (0–20 s) after start of the playback (trial value) by calculating the ratios of heart rate (bpm), RMSSD (ms), and RMSSD/SDNN between reference and trial value.

### Data Analysis

The experimental design resulted in a sample size of 384 trials (i.e., 24 farms × 4 experimental cows × 4 trials). However, five cows older than 10 years were excluded from data analysis to avoid the interference of age-dependent hearing impairment (*presbycusis*) with experimental treatments. One cow had to be excluded from data analysis due to technical problems and another cow due to not feeding at all. In eight trials (five cows on five farms), cows did not start feeding again after a playback exposure. In these cases, we had to quit the session. All these cows had no or very little experience with wearing a bell. Technical problems with video recording occurred in another seven trials. Thus, the total sample size was 89 cows on 24 farms in 341 trials.

Statistical analyses were performed in R 3.3.1 and 3.3.2 (R Core Team 2016, 43). We used the agreement package (51) to check the intraobserver agreement concerning the latency to the first reaction. To adequately reflect dependencies in the experimental design (nesting, repeated measurements), linear mixed-effects models were used to evaluate the latency to the first reaction and heartbeat measurements with the lmer methods from “lme4” and “lmerTest” packages, respectively (52). The occurrence of leaving the feeding rack was analyzed using a generalized linear mixed-effects model [glmer method from package “lme4” (53)]. Here, we used odds ratios [exponential function of the regression coefficient associated with a one-unit increase in the exposure (54)] to additionally quantify the effect sizes.

Full models consisted of the fixed-effects “bell experience” (factor with two levels: experienced, inexperienced), “earplugs” (factor with two levels: with, without), and “amplitude” (factor with two levels: 65 and 85 dB) and all possible interactions. Models were reduced in a stepwise backward procedure. *P* > 0.05 was used as criterion for exclusion of non-significant interactions. *P* values were calculated based on likelihood ratio tests. Trial nested in individual identity nested in farm served as random effects. To satisfy model assumptions, heartbeat parameters were log transformed.

## Results

In the figures, descriptive data and model estimations are shown; in the text, model estimations and odds ratios were used to interpret the results.

### Latency to the First Reaction

The intraobserver agreement for the assessment of the latency to the first reaction was good, with a concordance coefficient of 0.91.

**Figure 4:**
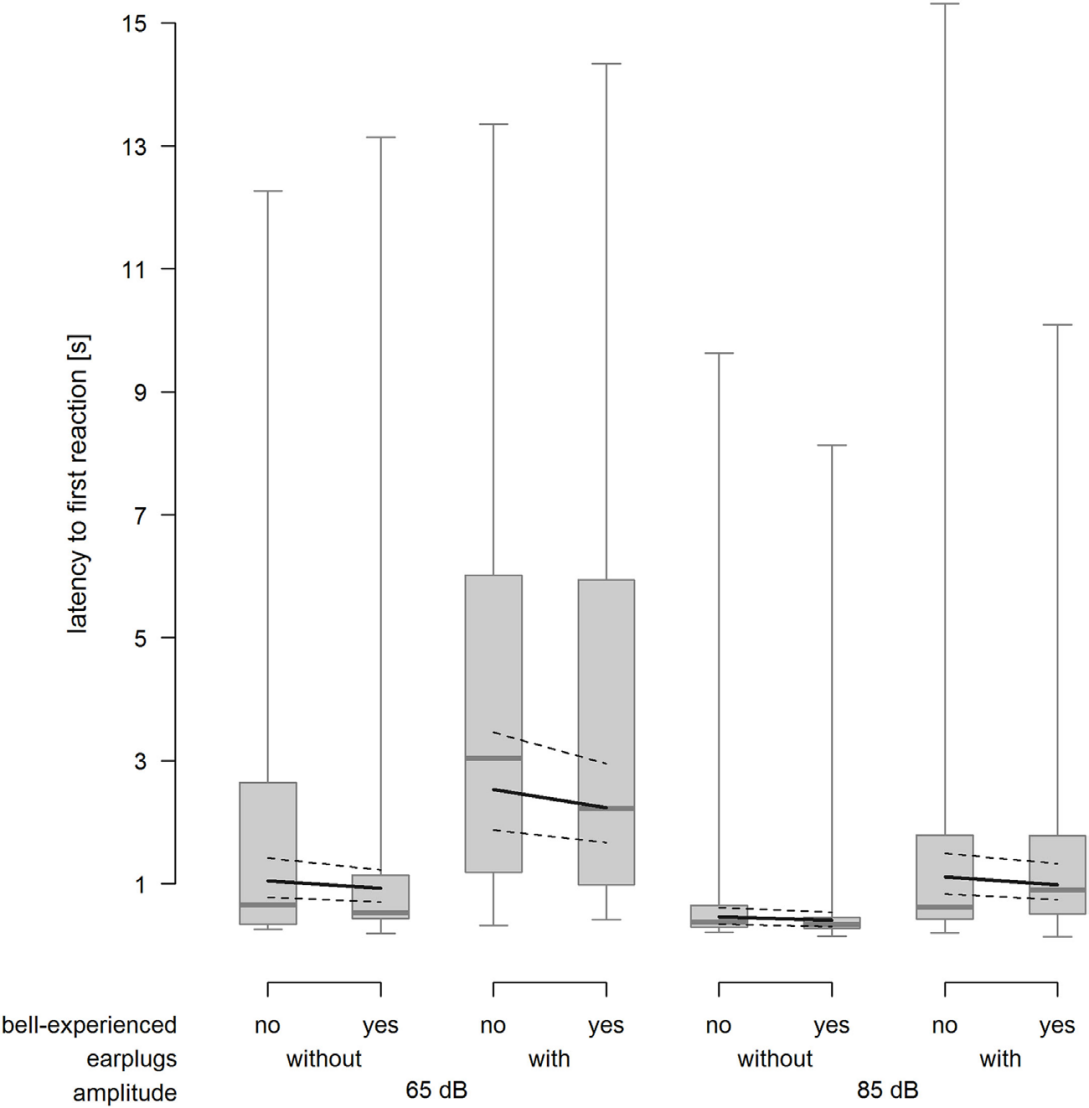
Latency to the first reaction 15 s after onset of the playback stimulus depending on bell-experienced (no, yes), earplugs (without, with) and amplitude (65, 85 dB). Descriptive data are presented as box plots indicating observed median, first and third quartiles, and absolute range of data. Solid lines show the model estimation and dotted lines show the lower and upper 95% confidence intervals.

As expected, cows reacted faster when exposed to the 85-dB stimulus compared with the 65-dB stimulus (*F*_1,212.1_ = 56.65, *P* < 0.001) and slower when equipped with earplugs (*F*_1,214.6_ = 65.05, *P* < 0.001). Bell experience did not affect the latency to the first reaction (*F*_1,24.5_ = 0.5, *P* = 0.486, Figure 4).

### Avoidance

**Figure 5:**
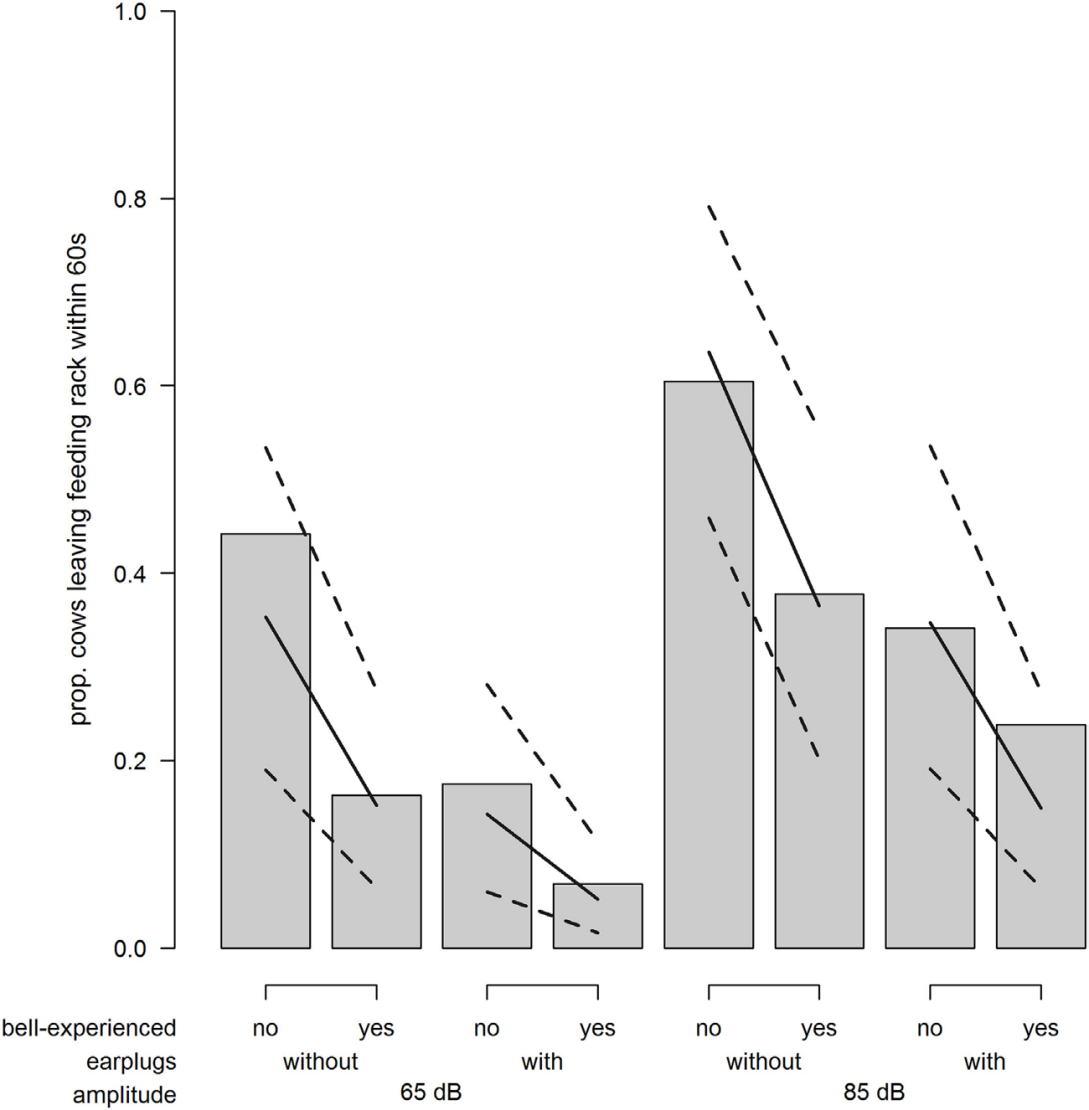
Avoidance. Proportion of cows leaving the feeding rack within 60 s after playback depending on bell-experienced (no, yes), earplugs (without, with), and amplitude (65, 85 dB). Solid lines show the model estimation, and dotted lines show the lower and upper 95% confidence intervals.

The probability for a cow to leave the feeding rack within 60 s after exposure to the sound stimulus was strongly increased when cows were exposed to 85 dB compared with 65 dB (odds ratio = 3.20; χ^2^ = 17.29; *P* < 0.001; Figure 5). Similarly, the probability to leave the feeding rack was reduced when wearing earplugs (odds ratio = 0.30; χ^2^ = 17.63; *P* < 0.001; Figure 5) and when cows were bell experienced (odds ratio = 0.33; χ^2^ = 4.92; *P* = 0.027; Figure 5).

### Heartbeat Measurements

In the description of the results of heartbeat measurements, a ratio >1 indicates that the trial value was greater than the reference value, and *vice versa* for a ratio <1.

**Figure 6:**
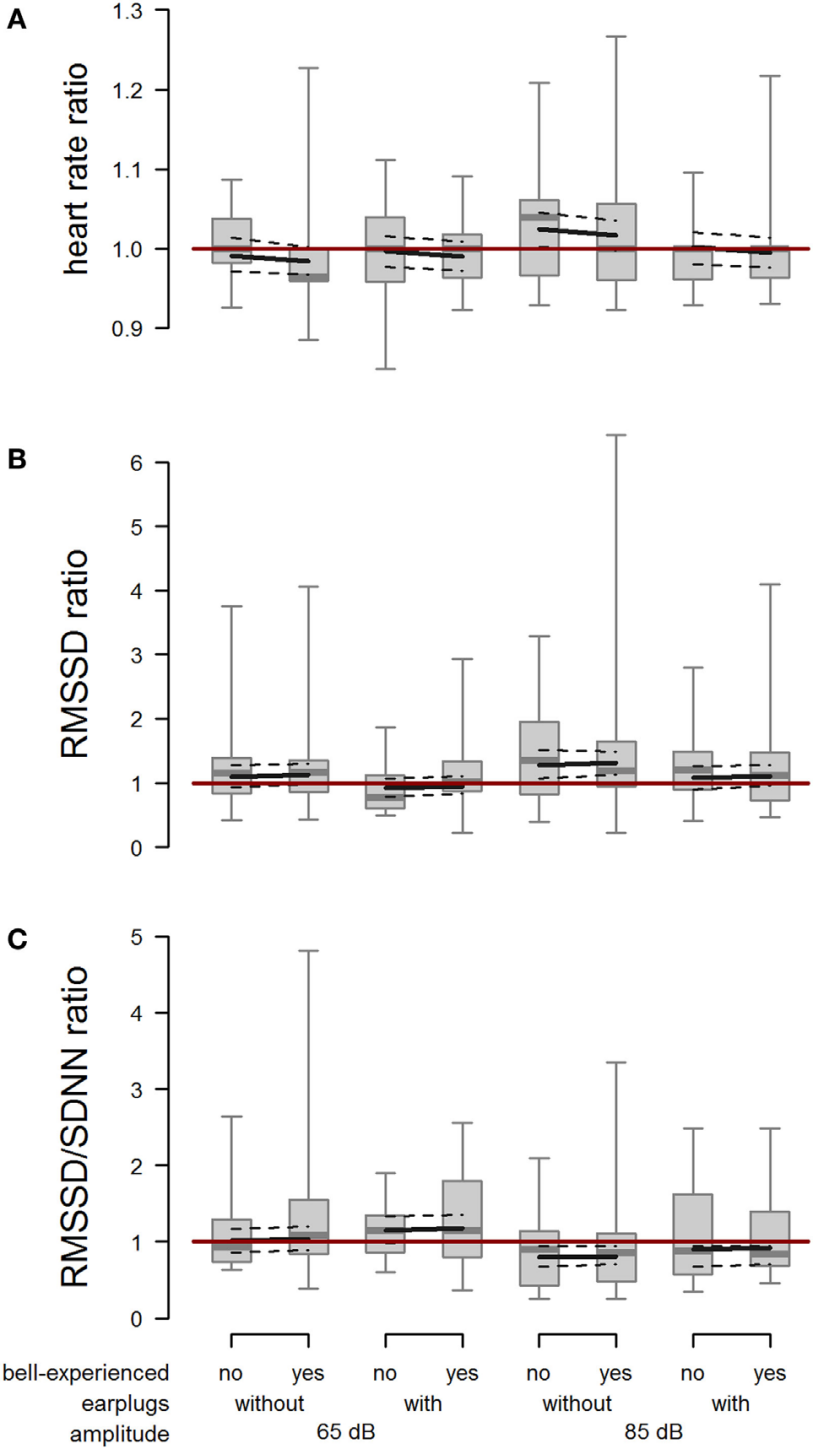
Heart rate, root mean square of successive difference (RMSSD), and RMSSD/standard deviation of heartbeats (SDNN). Ratio of **(A)** heart rate, **(B)** RMSSD, and **(C)** RMSSD/SDNN between playback and reference situation depending on bell-experienced (no, yes), earplugs (without, with), and amplitude (65, 85 dB). Descriptive data are presented as box plots indicating observed median, first and third quartiles, and absolute range of data. Solid lines show the model estimation, and dotted lines the lower and upper 95% confidence intervals. The dark red line represents the ratio value in case of identical values during trial and reference.

The mean absolute heart rate was 78.9 bpm (minimum: 54 bpm, maximum: 180 bpm) in the minute before and 79.5 bpm (minimum: 54 bpm, maximum: 180 bpm) in the minute after playback exposure and showed a large interindividual variability. When cows were exposed to 85 dB without earplugs, heart rate during the first 20 s after onset of the playback was increased compared with the baseline heart rate (amplitude × earplugs: *F*_1,100.1_ = 3.99; *P* = 0.048; Figure 6A). We found no effect of bell experience (*F*_1,16.8_ = 0.44; *P* = 0.515) on heart rate response.

The RMSSD ratio following the playback at 85 dB was increased compared with the RMSSD ratio following the playback at 65 dB (*F*_1,102.8_ = 5.58; *P* = 0.020). Further, RMSSD ratio was lower when wearing earplugs than when not wearing earplugs (*F*_1,48.4_ = 6.22; *P* = 0.016). We found no effect of bell experience (*F*_1,56.0_ = 0.09; *P* = 0.769) on RMSSD response (Figure 6B).

The RMSSD/SDNN ratio was reduced when cows were exposed to 85 dB compared with 65 dB (*F*_1,107.2_ = 13.45; *P* < 0.001), and it was slightly increased by earplugs (*F*_1,53.2_ = 3.27; *P* = 0.076; Figure 6C). Again, no effect of bell experience was detectable (*F*_1,60.0_ = 0.07; *P* = 0.797).

## Discussion

The experimental setting in this study allowed an assessment of the cows’ reactions to acoustic stimuli as both amplitude and earplugs affected all outcome variables in a meaningful way. However, none of the cows seemed to have severe hearing impairment as earplugs diminished the reactions of the cows to the acoustic stimuli. Overall, the cows responded to the 85-dB stimulus stronger than to the 65-dB stimulus. Bell experience and earplugs reduced avoidance of the noise stimulus. The high-amplitude stimulus (85 dB) triggered a shorter latency to the first reaction and increased heart rate and avoidance than the low-amplitude stimulus (65 dB). With earplugs, the latency to the first reaction was longer, and the cows showed less avoidance of the sound stimulus. However, bell experience had no effect on the latency to the first reaction.

Our results correspond to previous studies that found noises with higher amplitudes (e.g., >85 dB) evoke stronger behavioral reactions than noises with lower amplitudes in farm animals. When exposed to a playback of background noise recorded in milking parlors and broadcast at 85 dB in a raceway, heifers showed faster transit times, indicating an escape reaction (55). Heifers exposed to playbacks of people shouting (86 dB) and metal-on-metal clanging (85 dB) moved more often compared to no noise (56). Talling et al. (57) and Geverink et al. (58) found aberrant behavior when sound levels were up to 85 dB in pigs. Further, pregnant ewes that were exposed to noises with amplitudes increasing from 45 to 95 dB reduced their feeding time and increased the time spent inactive (59). MacKenzie et al. (60) found that hens showed avoidance to high-intensity noises (90–110 dB). Consequently, in this study, a shorter latency to the first reaction and increased avoidance indicate that cows perceived the acoustic stimuli as aversive.

The amplitudes of cowbells that are traditionally used on Alpine pastures vary between 90 and 113 dB (1). However, we did not measure the amplitudes of the bells that the experienced cows were wearing during pasture season. It is likely that there were differences in the amplitudes of the bells worn by the experienced cows. If so, this might explain the wide variability of responses observed in the experienced cows to a certain extent.

Furthermore, the increased heart rate during the 85-dB stimulus without earplugs corresponds to previous studies that found noises with higher amplitudes evoking stronger cardiac reactions than noises with lower amplitudes in pigs and humans. Talling et al. (57) found that pigs had a higher heart rate when exposed to very loud (97 dB) compared with loud (85 dB) stimuli. In addition, a sudden loud sound (110 dB, 1–20 kHz, 0.15 s) evoked an immediate increase of heart rate in humans (29). In our study, changes in heart rate, RMSSD, and the ratio between RMSSD and SDNN were rather small (i.e., ratios varied little and were close to 1), thus the cardiac reaction following the playback has to be interpreted with caution. However, Désiré et al. (61, 62) showed that the sudden appearance of an object elicited an increase in heart rate in lambs most likely due to enhanced sympathetic activity, i.e., increased arousal. In addition, the exposure to a novel object elicited an increase in RMSSD, indicating an increased parasympathetic activity. In the current study, heart rate after the playback at 85 dB was increased most likely due to enhanced sympathetic activity, and RMSSD ratio was increased due to an increased parasympathetic activity. At the same time, RMSSD/SDNN ratio was reduced, indicating a stronger activation of the sympathetic branch, overall. Consequently, the pink noise stimulus might have been both sudden and novel (31, 63).

Cows avoided the playback stimulus less when wearing earplugs, or when their farm of origin used bells during the pasture season (bell experienced), which may reflect an altered acoustic perception of the playback stimulus due to routine bell exposure. Furthermore, only cows from farms that did not use bells for their cows did not start feeding again after being exposed to the playback. This may indicate that the playback was perceived as aversive by these cows. Although the experimental setting of our study did not allow us to conclusively assess the hearing capacity of the cows, a reduced reactivity to the acoustic stimuli due to hearing impairment of bell-experienced cows cannot be excluded completely. In humans, dogs and mice hearing loss has been shown to occur after they were exposed to noise with amplitudes similar to those of cowbells. Further, exposure time in these studies was similar to the time cows are exposed to bells while on pasture (3–5, 33). However, given the effect of the earplugs and the fact that all cows in our study showed a reaction to the playback in at least one trial, none of them seemed to have a severe hearing impairment.

Considering the experimental setting in retrospect, the pink noise stimulus might not have been ideal to assess the full hearing capacity of cows. Given that cows can hear sounds between 23 Hz and 35 kHz (10), a high-pass filter that blocks LFs and passes HFs (64), or a stimulus that contains only higher frequencies might be more suitable to test cows’ hearing capacity in future studies since hearing impairment is associated with reduced sensitivity to HFs. In addition, the 85-dB stimulus used in our study might have been too loud to detect subtle differences in hearing capacities. On the other hand, the amplitude used to elicit an acoustic startle response was more than 80 dB in other studies (12, 18, 19, 29, 31). Thus, inferring information about hearing capacity from a latency to the first reaction to an acoustic stimulus as a proxy for the induction of a startle response might partly be misleading when using a high-amplitude stimulus, and other behavioral indicators have to be found. Furthermore, the studies mentioned above (3–5, 33) used standardized clinical hearing tests (BAEP and ABR). BAEP are bioelectric waves that can be recorded within 10 ms after an auditory stimulus and are used to assess auditory function (65). Due to the influence of excessive muscle movements on the measurement, it is necessary for the subject to be motionless during the procedure (37). Clinically, therefore, sedation is needed when measuring BAEP in young children or animals. Accordingly, BAEP measurements need to be conducted in a veterinary hospital rather than on-farm to be able to monitor the animals more closely.

Altogether, we could not clinically assess the hearing capacity of bell-experienced cows. Nevertheless, our indicators showed that routine bell exposure led to a mitigation of the behavioral response to a novel acoustic stimulus. Overall, the cows responded to the 85-dB stimulus stronger than to the 65-dB stimulus and using cowbells with lower amplitudes might be advantageous.

## Conclusion

Our results demonstrated that acute exposure to the 85-dB pink noise stimulus triggered increased arousal and avoidance compared with the 65-dB stimulus. Heart rate and heart rate variability indicated increased sympathetic activation during the exposure to 85 dB compared with 65 dB. Bell experience and wearing earplugs led to a generally decreased avoidance of the stimulus compared with bell-inexperienced cows and with cows not wearing earplugs. This may reflect an altered acoustic perception of the playback stimulus due to noise habituation, a low-reactive animal in general, or impaired hearing capacity when routinely exposed to bells.

## Ethics Statement

Ethical approval to conduct the study was obtained from the Zurich Cantonal Veterinary Office, Switzerland (approval No. 77/2012).

## Author Contributions

Study design: JJ and EH. Data collection: JJ, SM, and AP. Data analysis: JJ and EH. Manuscript drafting: JJ, AP, and EH. Critical revisions of the manuscript: SM, AP, and EH. Final approval: JJ, SM, AP, and EH.

## Conflict of Interest Statement

The authors declare that the research was conducted in the absence of any commercial or financial relationships that could be construed as a potential conflict of interest.
